# Development of a novel *in vitro* assay to screen for neuroprotective drugs against iatrogenic neurite shortening

**DOI:** 10.1371/journal.pone.0248139

**Published:** 2021-03-10

**Authors:** Antón L. Martínez, José Brea, Mateo Barro, Xavier Monroy, Manuel Merlos, Javier Burgueño, María Isabel Loza

**Affiliations:** 1 BioFarma Research Group, Centro Singular de Investigación en Medicina Molecular y Enfermedades Crónicas (CIMUS), Universidade de Santiago de Compostela, Santiago de Compostela, Spain; 2 WeLab Barcelona, Barcelona, Spain; Medical University Innsbruck, AUSTRIA

## Abstract

This work tries to help overcome the lack of relevant translational screening assays, as a limitation for the identification of novel analgesics for neuropathic pain. Hyperexcitability and neurite shortening are common adverse effects of antiviral and antitumor drugs, leading to neuropathic pain. Now, as seen in the drug screening that we developed here, a high-content microscopy-based assay with immortalized dorsal root ganglia (DRG) neurons (differentiated F11 cells) allowed to identify drugs able to protect against the iatrogenic neurite shortening induced by the antitumor drug vincristine and the antiviral drug rilpivirine. We observed that vincristine and rilpivirine induced a significant reduction in the neurite length, which was reverted by α-lipoic acid. We had also evidenced protective effects of pregabalin and melatonin, acting through the α_2_δ-2 subunit of the voltage-dependent calcium channels and the MT_1_ receptor, respectively. Additionally, two hits originated from a previous primary screening aimed to detect inhibitors of hyperexcitability to inflammatory mediators in DRG neurons (nitrendipine and felodipine) also prevented neurite shortening in our model. In summary, in this work we developed a novel secondary assay for identifying hits with neuroprotective effect against iatrogenic neurite shortening, consistent with the anti-hyperexcitability action previously tested: highlighting nitrendipine and felodipine against iatrogenic damage in DRG neurons.

## Introduction

Neuropathic pain is caused by an injury affecting either the central or the peripheral nervous system. It is characterized by spontaneous pain generated by non-painful stimuli (allodynia) or an exaggerated response to painful stimuli (hyperalgesia) [1]. Many etiologies, including diabetes, alcoholism, genetic diseases, metabolopathies, or infections have been implicated for the pathogenesis of this syndrome [2], along with the iatrogenic adverse effects of several drugs on the peripheral sensory neurons. These adverse effects comprise one of the major dose-limiting adverse effects of many antiviral and antitumor drugs [3, 4]. These drugs induce neuronal degeneration in the sensory nerves leading to denervation of the hands and feet producing sensory disturbances, including numbness and neuropathic pain that most often starts in the distal extremities in a glove-and-stocking distribution [5]. The conventional analgesic drugs, such as opioids or non-steroidal anti-inflammatory drugs (NSAIDs), are insufficient to alleviate neuropathic pain, and the relief from iatrogenic neuropathic pain remains a medical need because of the lack of effective analgesics for the treatment of this pathological condition. Consequently, drugs such as anticonvulsants (e.g., pregabalin or gabapentin), serotonin-specific reuptake inhibitors, and tricyclic antidepressants are being prescribed for the treatment of this condition, although these are all poorly effective [6].

Since the late 20^th^ century, many drug discovery programs have been centered on target-based assays and have evaluated the effect of molecules on previously identified targets. Thereby, new drugs for neuropathic pain share similar mechanisms of action and the current targets may not be relevant considering the actual pathogenesis occurring *in vivo* [7]. A phenotypic approach considering some disease engagement could imply an important advance in the development of novel analgesics to treat neuropathic pain as new drugs could be selected by their ability to alleviate translational attributes of that disease [8].

In a previous study, we developed a novel *in vitro* primary phenotypic high-throughput screening (HTS) assay based on hyperexcitability induced by inflammatory mediators in the sensory nerves on the F11 cell line, an immortalized dorsal root ganglia (DRG) neuronal cell line. Using this approach we discovered five hits (protriptyline, nicardipine, nimodipine, felodipine and nitrendipine) from the Prestwick library [9]. Here, we aimed to develop another phenotypic *in vitro* assay, in this case a high-content screening (HCS) secondary assay for evaluating the effect of drugs on the iatrogenic adverse effects (neurites shortening) of antivirals and antitumor agents on the immortalized DRG cell line F11. The antiviral rilpivirine and the antitumor agent vincristine were selected as they are prototypical drugs producing neuropathic pain in humans [10]. So, we aimed to propose a translational screening methodology to detect new drugs potentially useful for the treatment of iatrogenic neuropathic pain, acting both to amend excitability and neurite shortening induced by antitumor and antiviral treatments.

## Materials and methods

### Reagents

Vincristine (1257; Tocris, Bristol, United Kingdom), paclitaxel (1097; Tocris), rilpivirine (HY-10574; Haoyuan Chemexpress, Shanghai, China), and melatonin (M5250; Sigma-Aldrich, Madrid, Spain) were stored in dimethylsulfoxide (DMSO; D8418; Sigma-Aldrich) at a stock concentration of 10 mM. (±)-α-Lipoic acid (T1395; Sigma-Aldrich) was stored in ethanol (MERC1.00983.1000; VWR, West Chester, PA, USA) at a stock concentration of 100 mM. Pregabalin (3775; Tocris) was stored in Hank’s Balanced Salt Solution (HBSS) (pH 7.4) at a concentration of 10 mM. The stock compounds from Prestwick^®^ Chemical Library were dissolved in DMSO at a concentration of 10 mM and added to an intermediate 96-well plate by using an Echo liquid handler (Labcyte Inc., Cannock Chase, Staffordshire, United Kingdom) to a final concentration of 10 μM in a differentiation medium in the cell plate.

### Cell culture

F11 cells (08062601; ECCAC, Salisbury, England, UK) were grown in Dulbecco’s Modified Eagle’s Medium without sodium pyruvate (DMEM; D5671; Sigma-Aldrich) supplemented with 10% (v/v) non-dialyzed fetal calf serum (F9665; Sigma-Aldrich), 100 IU/ml penicillin and 100 μg/ml streptomycin (P0781; Sigma-Aldrich), and 2 mM glutamine (G7513; Sigma-Aldrich) in a humidified atmosphere containing 5% carbon dioxide, at 37 °C. F11 cells were routinely checked for mycoplasma contamination and tested negative.

### Immunofluorescence assays

Cells (80% confluence) were plated on clear-bottom 96-well plates (6005558; Perkin-Elmer, Madrid, Spain) previously treated with 30 μg/ml poly-D-lysine (P6407; Sigma-Aldrich). The cells were seeded in 50 μl of culture medium at a density of 7,500/well. After 24 h, the culture medium was replaced with a differentiation medium containing 100 IU/ml penicillin and 100 μg/ml streptomycin, 2 mM glutamine, 1 mM N^6^,2′-O-dibutyryladenosine 3′,5′-cyclic monophosphate (dibutyryl-cAMP; sc-201567; Santa Cruz Biotechnologies, Heidelberg; Germany), 30 μM forskolin (sc-3562; Santa Cruz Biotechnologies), and 0.5% dialyzed fetal calf serum (FCS_d_; F0392; Sigma-Aldrich) in Dulbecco’s Modified Eagle’s Medium without sodium pyruvate. After 72 h of differentiation, the cells were exposed to 1 nM vincristine, 100 nM rilpivirine, 1 μM paclitaxel, and to the indicated drugs whose effects were tested after dissolving in 50 μL of the differentiation medium. After 24 h, the medium was replaced with a fresh differentiation medium.

After 72 h of medium replacement, the F11 cells were fixed for 20 min with 4% paraformaldehyde (158127; Sigma-Aldrich) in HBSS (pH 7.4) at 4 °C. The cells were then washed twice with HBSS before permeabilizing with a blocking buffer containing 5% bovine serum albumin (BSA) (10775835001; Sigma-Aldrich) and 0.1% Triton X-100 (T8787; Sigma-Aldrich) in HBSS for 30 min at room temperature. The cells were then stained for 1 h with 1:500 dilution of Alexa 488 dye-conjugated anti-ß-tubulin mouse antibody (558605; Becton & Dickinson Biosciences, San Agustín de Guadalix, Madrid, Spain) and 2.5 μM nuclear stain DRAQ5 (108410; Abcam, Cambridge, UK). An Operetta High-Content Imaging System (Perkin-Elmer, Tres Cantos, Madrid, Spain) was used to capture all the fluorescence and bright field images (12 fields/well, 20X). The image analyses were performed with the Harmony High Content Imaging and Analysis software (Perkin-Elmer). At least four independent sets of experiments, each with at least six replicates, were performed.

### Real-time RT-PCR

Cells were seeded and differentiated on 6-well plates (CLS3516; Sigma-Aldrich) at a concentration of 250,000 cells/well. After 72 h of differentiation, the cells were exposed to 1 nM vincristine, 1 μM paclitaxel, and 100 nM rilpivirine. After 24 h, the total RNA was extracted using the RNeasy Plus Mini kit (741006; Qiagen, Las Matas, Madrid, Spain). The quality and quantity of the eluted RNA samples were verified by a NanoDrop spectrophotometer (Thermo Fisher Scientific, Alcobendas, Madrid, Spain). The total RNA (10 ng) was reverse transcribed to single-stranded cDNA using the express One-Step Superscript qRT-PCR kit (11781200; Invitrogen, Carlsbad, CA, USA). The real-time PCR analysis was performed with individual TaqMan gene expression assays in a QuantStudio 12K Flex reader (Life Technologies, Carlsbad, CA, USA) using standard experimental conditions designed by the manufacturer. The individual assay identification numbers were as follows: *Mtnr1a*: Mm00434999_m1, *Mtnr1b*: Mm00467511_m1, *Cacna2d1*: Mm01135167_m1, and *Cacna2d2*: Mm00457825_m1. All templates were analyzed in triplicate and the quantification cycle (C_q_) value of each gene was normalized to *36b4* according to ΔC_q_ = C_q_ (examined gene) − Cq (*36b4*).

### Data analyses

The maximum neurite length was measured employing Harmony High Content Imaging and Analysis software (Perkin-Elmer). The results were normalized to the maximum neurite length of control differentiated cells. The data obtained in the immunofluorescence assays were compared by Student’s *t*-test using GraphPad Prism^®^ 6.0 software (GraphPad, La Jolla, CA, USA), with the exception of data of the effect of the hits from Prestwick Chemical Library, that were compared by ANOVA followed by Dunett’s post-hoc test employing SPSS statistical software package version 25.0 (IBM Corporation, Armonk, NY, USA). The differences were considered statistically significant at p<0.05.

## Results

### A new *in vitro* phenotypic assay allowed the evaluation of iatrogenic neurite shortening

**Fig 1:**
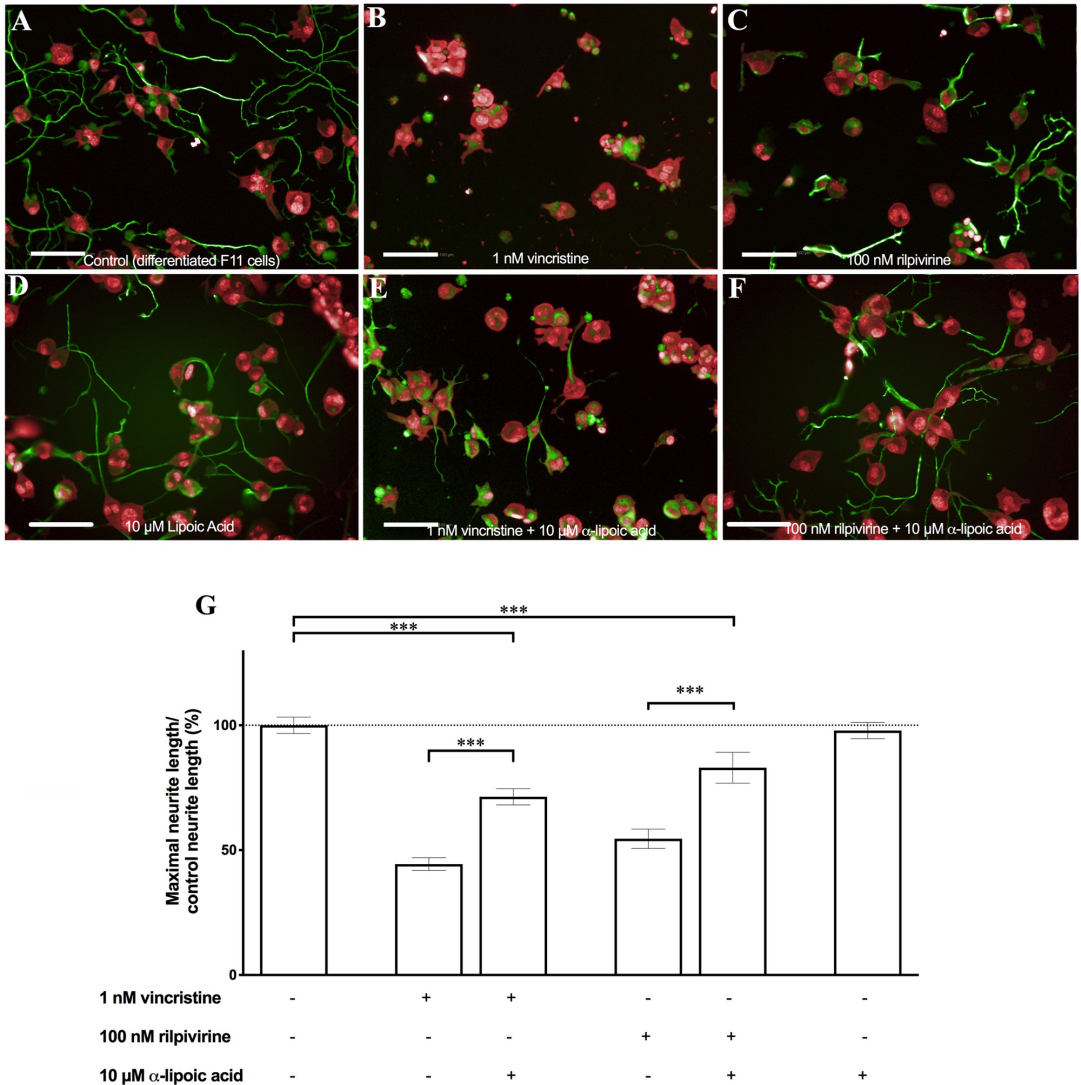
α-Lipoic acid induced a significant reversion of neurite shortening induced by vincristine and rilpivirine. The representative images of (A) differentiated F11 cells, (B) differentiated F11 cells exposed to 1 nM vincristine, and (C) to 100 nM rilpivirine are shown. The effects of 10 μM α-lipoic acid (D) on differentiated F11 cells and on the differentiated F11 cells exposed to (E) 1 nM vincristine and (F) 100 nM rilpivirine are shown. The images are representative of four assays each with six replicates (scale bar, 100 μm). (G) The maximal average length of the neurites quantified by image analysis in all the conditions tested is shown. The values represent the mean ± S.E.M. (n = 4, measurements performed in six replicates). ***p<0.001 (Student’s *t*-test).

The antitumor drug vincristine and the antiviral drug rilpivirine induced neurite shortening when administered alone (Fig 1B and 1C) as revealed by comparison with the control differentiated cells (Fig 1A). α-Lipoic acid (10 μM) was used as a positive control drug because it was previously described as a protective compound against the toxicity of neurotoxic drugs [11]. Despite the fact that it did not induced changes in neurite length by itself (Fig 1D), we observed in our assay that when it was employed in combination with vincristine and rilpivirine, it exerted a significant protective effect against the iatrogenic reduction of the neurite length caused by both vincristine and rilpivirine (Fig 1E–1G) (p<0.001, Student’s *t*-test).

### Pregabalin and melatonin showed protective effects against neurite shortening induced by antitumor and antiviral drugs in our assay

**Fig 2:**
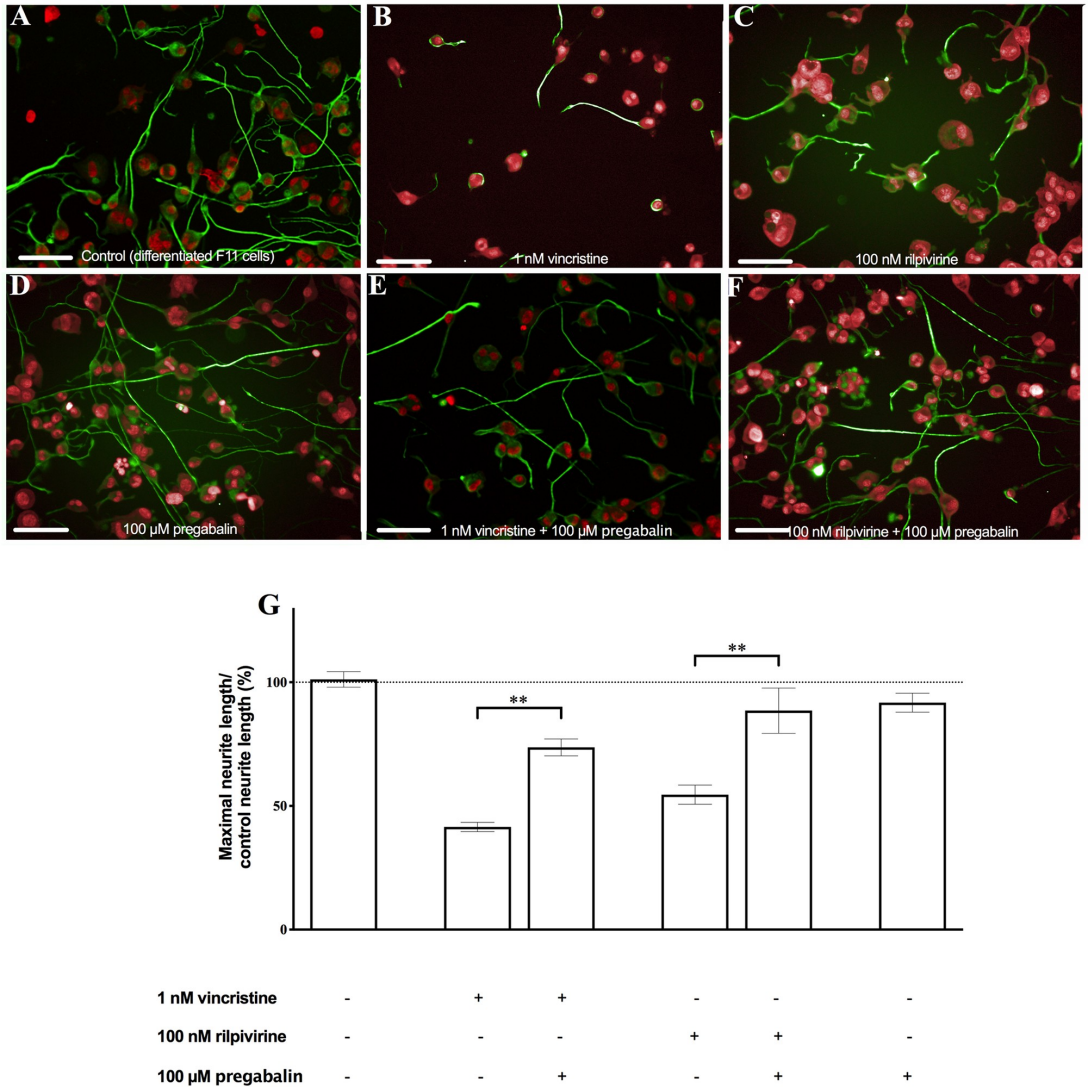
Pregabalin induced a significant reversion of neurite shortening induced by vincristine and rilpivirine. The representative images of (A) control differentiated F11 cells, of the effect of (B) 1 nM vincristine and (C) 100 nM rilpivirine on differentiated F11 cells, and (D) of the effect of 100 μM pregabalin on the differentiated F11 cells and on differentiated F11 cells exposed to (E) 1 nM vincristine and (F) 100 nM rilpivirine, are shown. The images are representative of four assays each with six replicates (scale bar, 100 μm). (G) The average length of the neurites quantified by image analysis in all the conditions tested is shown. The values represent the mean ± S.E.M. (n = 4, measurements performed in six replicates). **p<0.01 (Student’s *t*-test).

We checked whether two described drugs used for the treatment of neuropathic pain were protective against neurite shortening induced by the antitumor and antiviral drugs in our model. We exposed the differentiated F11 cells to a combination of 100 μM pregabalin with 1 nM vincristine or 100 nM rilpivirine. Pregabalin, a drug with an approved indication for the treatment of neuropathic pain [12], did not induce significative changes in neurite length by itself (Fig 2D), but induced a significant reversion of neurite shortening caused by the antiviral drug rilpivirine and the antitumor drug vincristine (Fig 2A–2G) (p<0.01, Student’s *t*-test).

**Fig 3:**
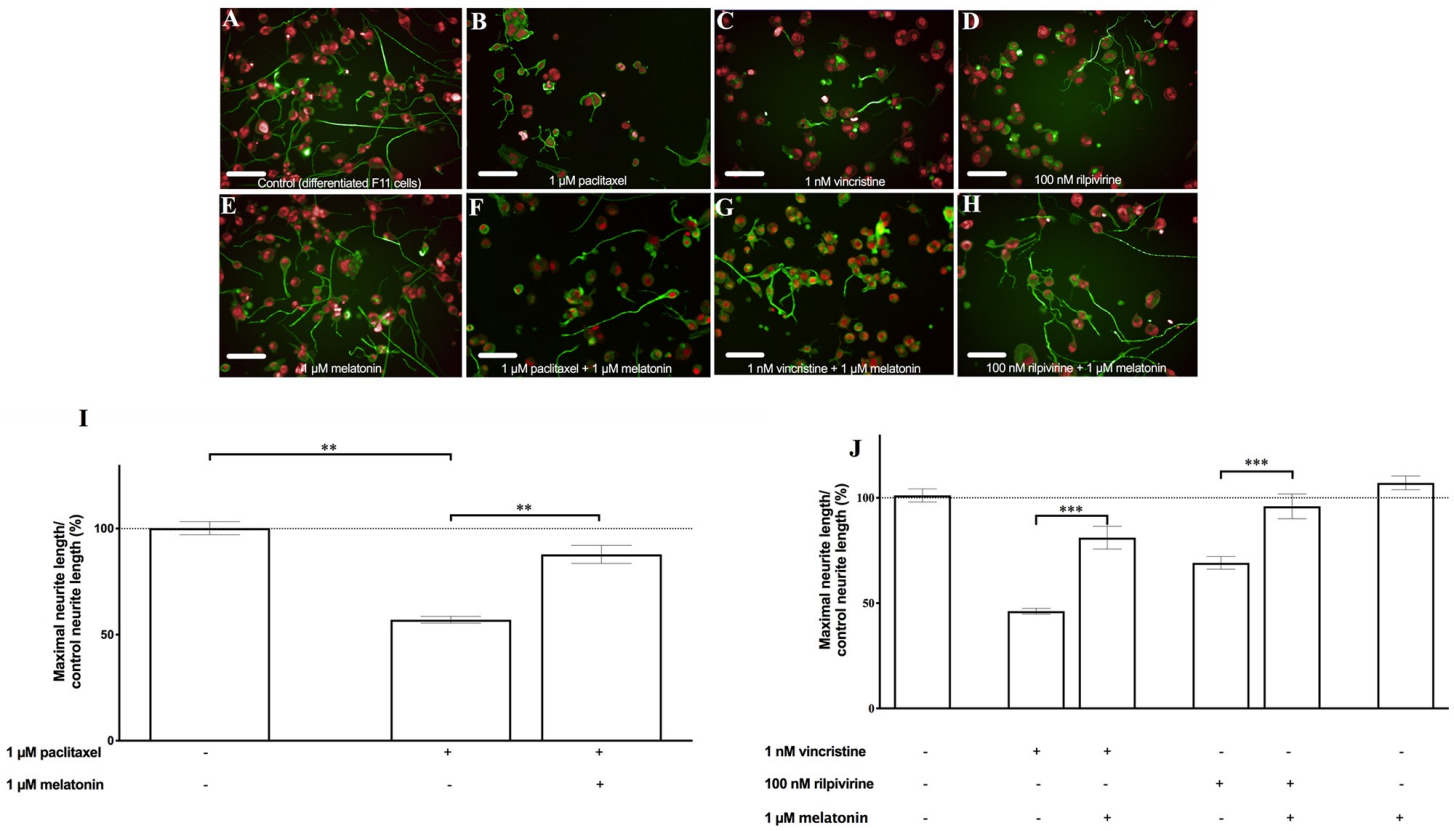
Melatonin induced a significant reversion of neurite shortening induced by paclitaxel, vincristine, and rilpivirine. Representative images of (A) control F11 differentiated cells, of the effect of (B) 1 μM paclitaxel, (C) 1 nM vincristine and (D) 100 nM rilpivirine on differentiated F11 cells and of (E) the effect of 1 μM melatonin on differentiated F11 cells and on differentiated F11 cells exposed to (F) 1 μM paclitaxel, (G) 1nM vincristine and (H) 100 nM rilpivirine are shown. The images are representative of four assays each with six replicates (scale bar, 100 μm). (I) Average length of the neurites quantified by image analysis in all the conditions tested is shown. The values represent the mean ± S.E.M. (n = 4, measurements performed in six replicates). *p<0.01 (Student’s *t*-test). (J) Graph depicting the average length of the neurites after treatment with 1 μM melatonin on the differentiated F11 cells exposed to 1 nM vincristine and 100 nM rilpivirine. The values represent the mean ± S.E.M. (n = 4, measurements performed in six replicates). ***p<0.01 (Student’s *t*-test).

To assess whether the model was valid for the detection of the protective effect of drugs reported to be effective in iatrogenic neuropathic pain, but not approved for the indication, we selected melatonin. Melatonin is reported to revert neuronal toxicity produced by the antitumor drug paclitaxel [13]. First, we exposed the differentiated F11 cells to 1 μM melatonin and 1 μM paclitaxel to assess if we could counteract neurite shrinkage induced by paclitaxel. We showed that 1 μM melatonin exerted a significant protective effect against neurite shortening elicited by 1 μM paclitaxel in our model (Fig 3A, 3B, 3F and 3I) (p<0.01, Student’s *t*-test), despite the fact that it did not induce significant changes in neurite length by itself (Fig 3E). To assess the validity of our approach, we also tested melatonin against the reduction of neurite length caused by vincristine and rilpivirine. We found that melatonin exerted a significant reversion of neurite length induced by both agents, reinforcing the validity of our approach (Fig 3C, 3D, 3G, 3H and 3J) (p<0.001, Student’s *t*-test).

### α_2_δ-2 subunit of the voltage-dependent calcium channels and MT_1_ receptor mRNAs were expressed in the differentiated F11 cells

Pregabalin is known to bind with similar affinity to both α_2_δ-1 and α_2_δ-2 subunits of the voltage-dependent calcium channels [14]. In order to ascertain the contribution of each target, we assessed the expression of the α_2_δ-1 and α_2_δ-2 subunits of the voltage-dependent calcium channels in our cells. We found that in the differentiated F11 cells exposed to vincristine and rilpivirine *Cacna2d2*, the gene that encodes the α_2_δ-2 subunit of the voltage-dependent calcium channels, was expressed, while the levels of mRNA encoded by *Cacna2d1*, the gene that encodes the α_2_δ-1 subunit of the voltage-dependent calcium, were undetectable (S1A Fig).

Melatonin exerts its effects acting through MT_1_ and MT_2_ receptors. In order to check the relative contribution of each target in the observed effect, we evaluated the expression of MT_1_ and MT_2_ receptors in our model. We found that in the differentiated F11 cells exposed to vincristine, paclitaxel, and rilpivirine, *Mtnr1a*, the gene that encodes the melatonin MT_1_ receptor, was expressed, whereas the levels of mRNA encoded by *Mtnr1b*, the gene that encodes the melatonin MT_2_ receptor, were undetectable (S1B Fig).

### Felodipine and nitrendipine reversed neurite shortening

**Fig 4:**
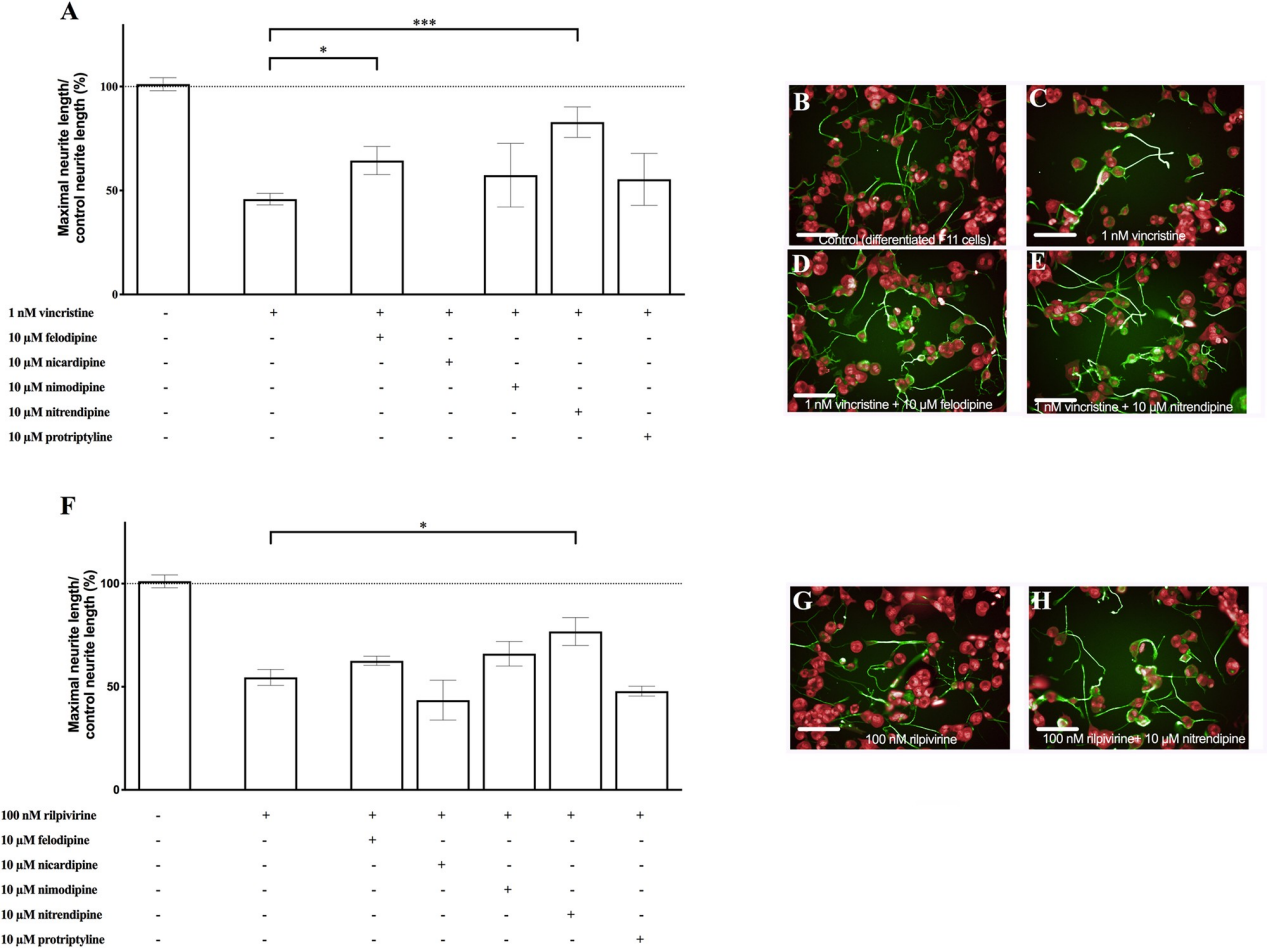
Felodipine and nitrendipine demonstrated activity counteracting vincristine-induced neurite shortening and nitrendipine protected against rilpivirine-induced neurite shortening in the differentiated F11 cells. (A) Graph showing the average length of the neurites after treatment with five hits from the Prestwick chemical library on the differentiated F11 cells exposed to 1 nM vincristine. The values represent the mean ± S.E.M. (n = 4, measurements performed in six replicates). *p<0.05, ***p<0.001 (ANOVA test followed by Dunett’s post-hoc analysis). Representative images of (B) control differentiated F11 cells, (C) differentiated F11 cells exposed to 1 nM vincristine and (D) to 1 nM vincristine and 10 μM felodipine, and to (E) 1 nM vincristine and 10 μM nitrendipine. (F) Graph showing the average length of the neurites after treatment with five hits from the Prestwick chemical library on the differentiated F11 cells exposed to 100 nM rilpivirine. The values represent the mean ± S.E.M. (n = 4, measurements performed in six replicates). *p<0.05 (ANOVA test followed by Dunett’s post-hoc analysis). Representative image of the effect of (G) 100 nM rilpivirine and of (H) 100 nM rilpivirine and 10 μM nitrendipine on the differentiated F11 cells. The image is representative of four assays each with six replicates (scale bar, 100 μm).

In order to validate the new assay for the discovery of the novel protective drugs against iatrogenic neurite shortening, we checked the effects of five hits from the Prestwick chemical library that previously showed an effect in reverting the hyperexcitability elicited by the inflammatory mediators [9]. From this list of five, felodipine and nitrendipine also significantly counteracted neurite shortening in the differentiated F11 cells exposed to vincristine (Fig 4A–4E) (p<0.05 for felodipine and p<0.001 for nitrendipine; ANOVA test followed by Dunett’s post-hoc analysis). Nitrendipine, also significantly protected against neurite shortening in the differentiated F11 cells induced by rilpivirine in differentiated F11 cells (Fig 4F–4H) (p<0.05, ANOVA test followed by Dunett’s post-hoc analysis).

## Discussion

The major finding of this work was the validation of two hits for iatrogenic neuropathic pain by a novel phenotypic assay employing a translational model. There is a lack of translational *in vitro* phenotypic screening assays for assessing drugs potentially useful for the treatment of neuropathic pain. Thus, we focused this study on the development of a new model for the identification of analgesic drugs based on their neuroprotective potential. Previously, we had identified five novel hits from a phenotypic screening assay that counteracted the hyperexcitability of the immortalized DRG neurons after exposing to inflammatory mediators [9]. Now, we have developed a complementary assay for new drugs on the neuronal damage present in the neuropathy.

Several drugs have been described to elicit iatrogenic neuropathic pain as a result of neurite shortening in the DRG neurons as an adverse effect [15]. It is the case of the neurite shortening effect of rilpivirine and vincristine in the immortalized DRG neuron-like cell line F11. This cell line has shown to retain many features of the native DRG neurons, including the synthesis of neurotransmitters and the expression of neuropeptides, chemokines, opioid receptors, and voltage-gated calcium channels. This explains why the F11 cells are used as surrogates for the primary DRG neurons in many studies, including those involving the neurobiology of neuropathic pain [16, 17]. Up to date the *in vitro* models available for measuring neurite shortening employed primary DRG neurons [18], however they had several limitations, like the low throughput of the assays and the use of animals for neuron obtention. These issues are avoided by employing differentiated F11 cells, since they are an unlimited source of cells allowing High Content Imaging assays, their easy differentiation, as well as the fact that it avoids the presence of non-neuronal cells that may interfere with neuronal signals [19].

Vincristine is an antitumor drug that inhibits microtubule depolymerization inducing mitotic arrest [20]. Its toxicity is related to mitochondrial dysfunction. An increase in the intracellular calcium level leads to axon degeneration in the DRG neurons [21–23]. Rilpivirine is an antiviral drug that belongs to the group of reverse transcriptase inhibitors. One of the most relevant adverse effects of this group is neuropathic pain attributed to damages caused to the DRG neurons [24–26].

In this new model, we were able to verify a clear neurite shortening induced by both the antitumor drug vincristine at a concentration of 1 nM and the antiviral rilpivirine at a concentration of 100 nM. For this purpose, we set up a microscopy assay employing a high-content automated fluorescence microscope to quantify the reduction in the neurite length in the differentiated F11 cells grown on 96-well plates.

As a control, we used α-lipoic acid because it was described as a protective compound for patients treated with neurotoxic drugs [27]. α-Lipoic acid is an antioxidant that protects against reactive oxygen species-induced mitochondrial dysfunction [11]. In our study, 10 μM α-lipoic acid was used according to the plasma concentration achieved in clinical practice [28]. We observed that this compound protected against neurite shortening induced by both vincristine and rilpivirine in the differentiated F11 cells.

For evaluating the system predictiveness, we tested pregabalin and melatonin, two already described drugs for neuropathic pain. 100 μM pregabalin, as a first-line drug [12], significantly prevented neurite shortening induced by both 1 nM vincristine and 100 nM rilpivirine. This concentration was similar to therapeutic pregabalin blood levels described in several clinical trials [29].

It is described that pregabalin prevents hyperexcitability of the sensory neurons in patients with neuropathic pain through the inhibition of α_2_δ-1 and α_2_δ-2 subunits of the voltage-gated calcium channels [14]. So, we checked the expression of mRNA encoding both subunits. We observed that mRNA encoding the α_2_δ-2 subunit of the voltage-gated calcium channels was predominantly expressed while mRNA encoding α_2_δ-1 subunit expression was undetectable. These results suggest that the protective effects of pregabalin are mediated by its interaction with the α_2_δ-2 subunit. Indeed, Tedeschi et al. (2016) showed that the blockade of the α_2_δ-2 subunits by pregabalin promotes axonal growth both in the central and peripheral neurons [30].

Another antitumor drug that has been reported to induce DRG toxicity is paclitaxel [31]. Galley et al (2017) showed that paclitaxel increases the membrane potential and reduces the metabolic activity in the DRG cells by altering the mitochondrial function [13]. In the same work, the authors proved that melatonin limits paclitaxel toxicity both in a rodent model of paclitaxel-induced neuropathic pain and an *in vitro* assay evaluating the mitochondrial membrane potential [13].

In our study, we observed that paclitaxel leads to a reduction in the neurite length in the differentiated F11 cells and that this neurite shortening could be reverted by 1 μM melatonin as described by Galley et al. [13]. Moreover, we observed that 1 μM melatonin also protected against neurite shortening induced by both vincristine and rilpivirine. We observed that MT_1_ receptor is the predominant receptor for melatonin expressed in our system, which is in agreement with previous results on neuroprotective and antioxidant effects of melatonin acting on MT_1_ receptor [32]. Furthermore, it was previously shown by Kaneko et al. (2011) that MT_1_ receptor was related to neurogenesis [33].

In our lab, we had previously performed a screening through a novel phenotypic primary assay of the excitability in the F11 cell model and five hits with potential analgesic effects (felodipine, nitrendipine, nicardipine, nimodipine, and protriptyline) were detected from the Prestwick chemical library [9]. All those hits were already described as drugs that could be useful for relieving neuropathic pain [34, 35]. So, we also checked these compounds in the novel phenotypic assay. Felodipine and nitrendipine protected against the reduction in the neurite length induced by vincristine, while nitrendipine protected against the reduction in the neurite length induced by rilpivirine. Both felodipine and nitrendipine are L-type calcium channels inhibitors belonging to the chemical group 1,4-dihydropyridines [37]. Moreover, both compounds block calcium effluxes from mitochondria [36], having been described that the potency of nitrendipine is greater than the potency of felodipine [37], suggesting that their neuroprotective role may be driven by modulating mitochondrial calcium homeostasis. Indeed, the smaller effect of both compounds as protectors against neurite shortening elicited by rilpivirine compared to the protective effect induced by vincristine may be due to the fact that vincristine toxicity is related to a disbalance in calcium homeostasis in mitochondria [38], while rilpivirine effect on mitochondria is more modest [39, 40].

Although a neuroprotective action has been described for all the inactive compounds in our model, their mechanism of action is not related to the protection against neurite shortening. Thereby, nimodipine and nicardipine exert an anti-inflammatory action in microglia in neurodegenerative diseases, while felodipine enhances autophagy in the brain [41]. On the other hand, protriptyline has been described as a neuroprotective drug in Alzheimer disease but its action is related to the defense against the formation of amyloid aggregates [42]. This emphasizes that the neuroprotective effect of felodipine and nitrendipine comes from their different mechanism of action involving mitochondrial calcium homeostasis and discard that it would come from any non-specific side effect.

In summary, in this study we developed a phenotypic microscopy-based assay employing the DRG-like sensitive neurons against neurite shortening induced by vincristine and rilpivirine and validated by using the positive controls α-lipoic acid, pregabalin, and melatonin. This assay complements as a secondary screening our previously developed hyperexcitability assay, whereas the new neurite shortening assay is focused on the detection of compounds with neuroprotective properties. Taking into account the complexity of neuropathic pain, we believe that this type of complementary primary and secondary assays will help in advance towards the detection of useful analgesics for different etiologies of neuropathic pain. Finally, we found that felodipine and nitrendipine can behave as such potential analgesics with both anti-hyperexcitability and neuroprotective actions.

## Supporting information

S1 Fig Pregabalin and melatonin exert their neuroprotective effects on F11 cells through the α_2_δ-2 auxiliary subunit of the calcium voltage-gated channel and the MT_1_ receptor, respectively. (A) Expression of *Cacna2d1* and *Cacna2d2* mRNA, that encode α_2_δ-1 and α_2_δ-2 calcium voltage-gated channel subunits, respectively, in the differentiated F11 cells after exposure to 1 nM vincristine and 100 nM rilpivirine as compared to the control differentiated cells are shown. The values shown are the mean ± S.D. of ΔC_q_ (n = 2, measurements per triplicate). The *Cacna2d1* mRNA levels were undetectable. (B) Expression of *Mtnr1a* and *Mtnr1b* mRNA, that encode melatonin MT_1_ and MT_2_ receptors, respectively, in the differentiated F11 cells after exposure to 1 nM vincristine, 100 nM rilpivirine, and 1 μM paclitaxel as compared to the control differentiated cells. The data represent the mean ± S.D. of ΔC_q_ (n = 2, values per triplicate). The *Mtnr1b* mRNA levels were undetectable.
(TIF)
