# Supplementary figures and images for: Development of a novel in vitro assay to screen for neuroprotective drugs against iatrogenic neurite shortening
Source: PLoS One. 2021 Mar 10;16(3):e0248139. doi: 10.1371/journal.pone.0248139 (PMC7946280; doi:10.1371/journal.pone.0248139)

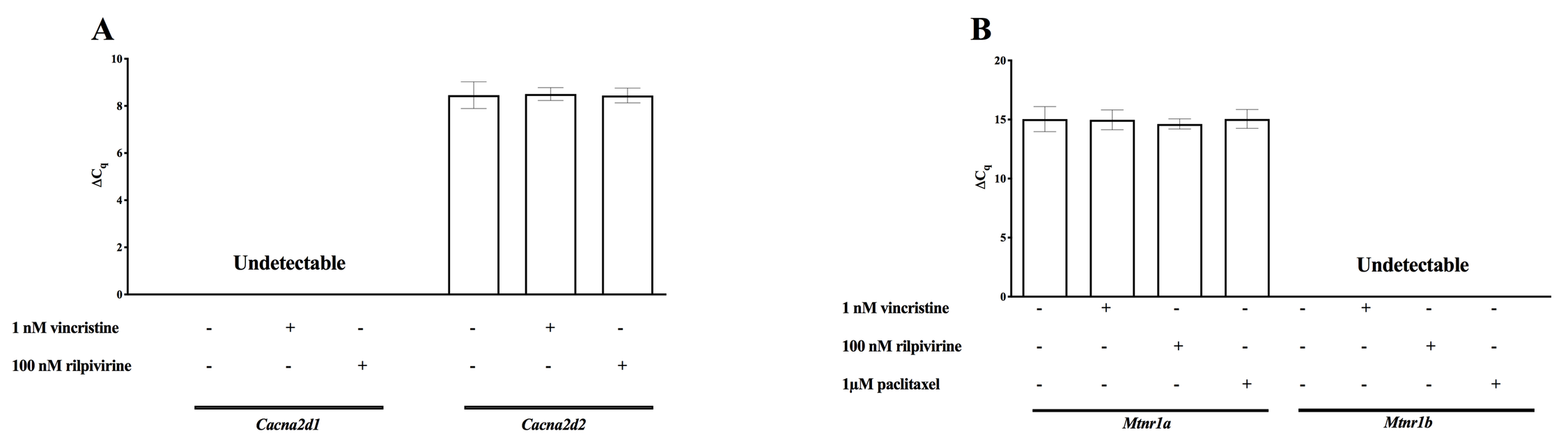

Supplement: S1 Fig — (A) Expression of Cacna2d1 and Cacna2d2 mRNA, that encode α2δ-1 and α2δ-2 calcium voltage-gated channel subunits, respectively, in the differentiated F11 cells after exposure to 1 nM vincristine and 100 nM rilpivirine as compared to the control differentiated cells are shown. The values shown are the mean ± S.D. of ΔCq (n = 2, measurements per triplicate). The Cacna2d1 mRNA levels were undetectable. (B) Expression of Mtnr1a and Mtnr1b mRNA, that encode melatonin MT1 and MT2 receptors, respectively, in the differentiated F11 cells after exposure to 1 nM vincristine, 100 nM rilpivirine, and 1 μM paclitaxel as compared to the control differentiated cells. The data represent the mean ± S.D. of ΔCq (n = 2, values per triplicate). The Mtnr1b mRNA levels were undetectable. (TIF) [file pone.0248139.s001.tif]
